# A 19-SNP coronary heart disease gene score profile in subjects with type 2 diabetes: the coronary heart disease risk in type 2 diabetes (CoRDia study) study baseline characteristics

**DOI:** 10.1186/s12933-016-0457-7

**Published:** 2016-10-03

**Authors:** Katherine E. Beaney, Claire E. Ward, Dauda A. S. Bappa, Nadine McGale, Anna K. Davies, Shashivadan P. Hirani, KaWah Li, Philip Howard, Dwaine R. Vance, Martin A. Crockard, John V. Lamont, Stanton Newman, Steve E. Humphries

**Affiliations:** 1 British Heart Foundation Laboratories, Centre for Cardiovascular Genetics, Institute of Cardiovascular Science, University College London, University Street, London, UK; 2 Molecular Diagnostics Group, Randox Laboratories Ltd, Crumlin, UK; 3 School of Health Sciences, City University London, Northampton Square, London, UK

**Keywords:** Type 2 diabetes, Coronary heart disease, Gene score, UKPDS score, Risk prediction

## Abstract

**Background:**

The coronary risk in diabetes (CoRDia) trial (n = 211) compares the effectiveness of usual diabetes care with a self-management intervention (SMI), with and without personalised risk information (including genetics), on clinical and behavioural outcomes. Here we present an assessment of randomisation, the cardiac risk genotyping assay, and the genetic characteristics of the recruits.

**Methods:**

Ten-year coronary heart disease (CHD) risk was calculated using the UKPDS score. Genetic CHD risk was determined by genotyping 19 single nucleotide polymorphisms (SNPs) using Randox’s Cardiac Risk Prediction Array and calculating a gene score (GS). Accuracy of the array was assessed by genotyping a subset of pre-genotyped samples (n = 185).

**Results:**

Overall, 10-year CHD risk ranged from 2–72 % but did not differ between the randomisation groups (p = 0.13). The array results were 99.8 % concordant with the pre-determined genotypes. The GS did not differ between the Caucasian participants in the CoRDia SMI plus risk group (n = 66) (p = 0.80) and a sample of UK healthy men (n = 1360). The GS was also associated with LDL-cholesterol (p = 0.05) and family history (p = 0.03) in a sample of UK healthy men (n = 1360).

**Conclusions:**

CHD risk is high in this group of T2D subjects. The risk array is an accurate genotyping assay, and is suitable for estimating an individual’s genetic CHD risk.

*Trial registration* This study has been registered at ClinicalTrials.gov; registration identifier NCT01891786

## Background

There has long been evidence from observational studies that those with type 2 diabetes (T2D) have an increased risk of coronary heart disease (CHD) [1] and recent genetic studies support a causal role for diabetes in CHD [2, 3]. Furthermore, there is evidence from randomised controlled trials that better glycaemic control results in better cardiovascular outcomes as well as reducing the risk of diabetic complications [4, 5].

Given the importance of diabetes management for cardiovascular health and evidence that patients often have difficulty following recommended behaviour (e.g. maintaining a balanced diet and regular exercise), self-management interventions (SMI) have been developed [6]. These structured group educational sessions led by trained facilitators allow patients to identify the difficulties they face in managing their diabetes with their peers, and use theory- and evidence-based techniques to enable patients to develop the skills to manage their health-related behaviours. The Coronary heart disease Risk in type 2 Diabetes (CoRDia) study was designed to investigate whether attendance at an SMI with and without receiving personalised CHD risk information (including conventional and genetic risk), resulted in improved glycaemic control and reduced CHD risk compared with usual care [7]. Ten-year risk of CHD was determined using the UKPDS score [8].

Gene scores can be used to provide an estimate of genetic risk for a particular phenotype. A 19 single nucleotide polymorphism (SNP) CHD gene score comprised of variants in risk loci identified by both candidate studies and genome-wide association studies (GWAS) has been found to be associated with CHD in those of Caucasian [9] and Afro-Caribbean ethnicity [10], although the relationship with CHD in those of South Asian ethnicity is unclear [9]. Furthermore, the gene score showed potential clinical utility in the UK population as in healthy UK subjects, when considering those who went up a risk category with the addition of the gene score to the Framingham risk score, a statistically significantly number were reclassified correctly. The gene score has not previously been tested in a group with type 2 diabetes.

Having collected the baseline clinical data, our first aim was to compare the conventional risk factor (CRF) data across the three CoRDia groups, and thus determine whether randomisation was effective. Our second aim was to examine the accuracy of genotyping performed using the Cardiac Risk Prediction array (Randox Laboratories Ltd, Crumlin, Co Antrim, UK) to examine the accuracy of the SNP score genotyping protocol. Finally, we sought to compare the genetic profile of the recruits with a sample of healthy UK men to assess whether the results observed in the CoRDia study are applicable to the general population and to assess whether the gene score was associated with any risk factors for CHD.

## Methods

### CoRDia

The protocol for the CoRDia study has been described in detail elsewhere [7]. Briefly, 211 patients with poorly controlled T2D [defined by a glycated haemoglobin (hba1c) level ≥ 6.45 %] were recruited from primary care in the East of England. Each participant was randomised into one of three arms; (i) usual care, (ii) SMI only or (iii) SMI plus personalised CHD risk (SMI plus risk results—SMI + RR). Sample size was based on a power calculation for the number of participants required to detect a 6.25 % difference in glycated haemoglobin levels (i.e. a clinically achievable reduction) between any of the study groups after follow-up of 12 months. This allows for both increased motivation in our intervention groups and thus a decrease in these group(s) compared to the control group (as we would hypothesise) but also that participants may be de-motived by knowledge of their personal CHD risk resulting in an increase compared to the control group. Personalised CHD risk was determined using the UKPDS risk score [8]. The UKPDS score includes age of T2D onset, sex, Afro-Caribbean ethnicity, total cholesterol, high density lipoprotein (HDL) cholesterol, systolic blood pressure, duration of diabetes, smoking and glycated haemoglobin, and calculates the risk of developing CHD in those with T2D in the next 10 years. A combined CHD risk score was determined for individuals in the SMI + RR group where the UKPDS score was combined with the 19 SNP CHD gene score [9]. The participants aged between 25 and 74 years and were of Caucasian, Afro-Caribbean, Asian Indian, mixed Caucasian/Afro-Caribbean or mixed Caucasian/Asian Indian ethnicity. When calculating the UKPDS score those of mixed Caucasian/Afro-Caribbean ethnicity were coded as non-Afro-Caribbean (ethnicity is a binary variable based on Afro-Caribbean ethnicity in the UKPDS score) as suggested by the developers of the UKPDS risk score. All participants were free of cardiovascular disease at baseline. Recruits into the SMI + RR arm consented to provide a saliva sample for analysis using the Oragene DNA Self-collection Kit (DNA Genotek Inc., Ontario, Canada). Collected samples were marked with a barcode and posted to the genetics laboratory at UCL. Samples were received within approximately one week. Ethical approval for this study has been granted by the East of England Research Ethics Committee (ref 12/EE/0437) and the study complied with the ethical principles underlying the Declaration of Helsinki. This study has been registered at ClinicalTrials.gov; registration identifier NCT01891786.

### Healthy subject comparison group

Healthy UK men were selected from the second Northwick Park Heart Study (NPHSII) [11], which is a prospective study of middle-aged Caucasian men recruited from general practices in the UK. Participants were free of cardiovascular disease at baseline. There was a median of 13.5 years follow-up. CHD was defined as acute myocardial infarction (MI), silent MI or having undergone coronary surgery. Family history of early CHD was collected as reported previously [12]. Complete genotype data was available for 1360 men. Ethical approval was given from the national research ethics service (NRES) Committee London-Central.

### Genotyping

Genotyping of NPHSII samples was performed on DNA extracted from blood and carried out using Taqman (Applied Biosystems, Life Technologies, Carlsbad, CA, USA) and KASPar (LGC, Teddington, UK) genotyping assays as well as restriction fragment length polymorphism (RFLP) analysis. For the CoRDia SMI + RR arm, saliva was collected using the Oragene-DNA OG-250 and DNA manually purified using DNA Genotek’s PrepIt-L2P DNA extraction kit (DNA Genotek Inc., Ontario, Canada). Individuals in the CoRDia SMI + RR arm and selected NPHSII samples (n = 185) were genotyped using the Cardiac Risk Prediction Array (Randox Laboratories Ltd, Crumlin, Co Antrim, UK) according to the manufacturer’s instructions. The Cardiac Risk Prediction Array is a multiplex SNP genotyping system which uses Randox’s Biochip Array Technology [13] to genotype 19 CHD risk SNPs. The protocol involves using multiplex PCR to amplify target DNA in an allele-specific manner. Amplicons are detected by hybridisation to spatially tethered probes on the biochip array surface. Each position on the biochip array corresponds to a specific allele and genotypes are determined using the Evidence Investigator Analyser. In some instances (e.g. for rare genotypes) Sanger sequencing was used to confirm the result.

### Statistical analysis

Statistical analysis was carried out using R version 3.1.2. The gene score was calculated as detailed previously [9]. Briefly, the number of risk alleles for a particular SNP was multiplied by the effect size pertaining to the association between that SNP and CHD (apart for rs1799983 which was treated in a recessive manner). The UKPDS score was calculated as described in [8]. For the CoRDia SMI + RR group the gene score was calculated and combined with the UKPDS score using a proprietary algorithm (Storegene, http://www.storegene.com/) to give a combined risk score. Briefly, to combine the gene score with the UKPDS score, the relative odds ratio determined by the gene score was converted to relative risk (incidence of CHD in this group was set at 0.3: calculated using data published by the British Heart Foundation [14]) and included as a term in the calculation of “q” (used to calculate the UKPDS score). Combined ten-year CHD risk could then be calculated by exponentiating the product of “q” and (1-duration of T2D^10)/1-duration of T2D) and subtracting it from one. Clinical and demographic variables were compared across the three randomisation groups using ANOVA for numerical data and Chi squared tests for categorical data. A difference of p ≤ 0.05 was taken as statistically significant.

## Results

**Fig. 1:**
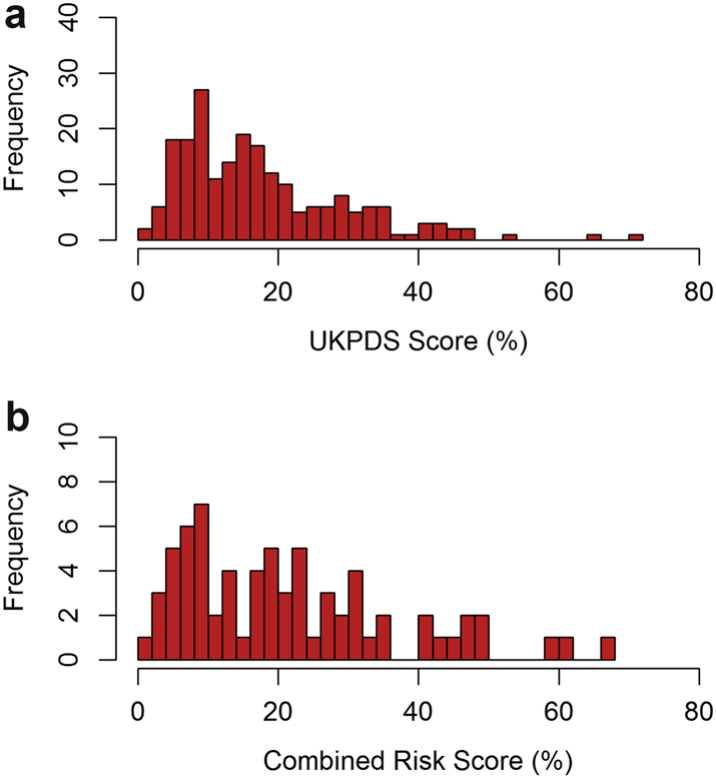
Distribution of **a** UKPDS risk score in CoRDia participants (n = 210*) and **b** Combined risk score (UKPDS plus genetic risk) in CoRDia SMI plus risk results group (n = 70)

The baseline characteristics of the participants in the CoRDia study are shown in Table 1. Of the T2D-CHD risk factors, only sex and HDL-cholesterol were found to differ between the three randomisation groups (p = 0.05). The 10-year risk of CHD in the whole of the CoRDia study (n = 211) ranged from 2–72 % as shown in Fig. 1a and did not differ significantly between the study groups (p = 0.13). Median UKPDS CHD risk in the CoRDia subjects (n = 211) was 14.89 % (interquartile range 8.58–23.53 %). For reference, the median Framingham CHD risk was 10.56 % (interquartile range 7.30–15.56 %) in the NPHSII cohort (n = 2948), although the CHD risks cannot be directly compared as they were calculated using different scores.

**Table 1:** Baseline characteristics of the CoRDia participants by randomisation group

Trait	CoRDia control group (n = 67)	CoRDia SMI only group (n = 74)	CoRDia SMI + RR group (n = 70)	p value
Age (years)	61.40 (10.08)	61.28 (9.11)	62.36 (7.42)	0.53
Sex (% female)	48 % (n = 33)	46 % (n = 40)	34 % (n = 24)	0.05
Ethnicity (% European)	92 % (n = 60)	92 % (n = 68)	94 % (n = 66)	0.65
Total cholesterol (mmol/l)^a^	4.18 (0.97)	4.36 (0.89)	4.29 (1.03)	0.55
HDL-cholesterol (mmol/l)^a^	1.27 (0.29)	1.23 (0.37)	1.16 (0.29)	0.05
Systolic blood pressure (mmHg)	133.93 (12.56)	134.36 (14.41)	133.44 (12.97)	0.97
Duration of T2D (years)^b^	5.60 (0–23)	5.60 (0–39)	6.33 (0–23)	0.38
Age of T2D onset (years)	55.00 (10.87)	54.99 (10.17)	54.90 (8.36)	0.95
Glycated haemoglobin (%)^a^	7.73 (1.29)	7.60 (1.03)	7.58 (0.99)	0.41
Smoking (n)	7 % (n = 5)	5 % (n = 4)	16 % (n = 11)	0.09
UKPDS risk score (%)^a^	13.40 (8.28–22.90)	14.36 (8.02–22.02)	17.25 (9.40–26.53)	0.13

The mean plus standard deviation is shown, where appropriate. Numeric variables were compared using ANOVA and categorical variables were compared using Chi squared tests

*HDL* high density lipoprotein, *T2D* type 2 diabetes, *SMI* self-management intervention, *RR* risk report

^a^Variable was log transformed. Geometric mean and approximate standard deviation are shown (except UKPDS risk score where the inter-quartile range is shown)

^b^Variable was square root transformed, means were transformed back and the range is shown

The saliva collection method and DNA extraction procedure was successful for all samples and the yield of DNA ranged from 5–100 µg. To validate the methodology, genotyping of a sub-sample (n = 185) from the NPHSII cohort of healthy UK subjects for the 19 SNPs was carried out using the Cardiac Risk Prediction Array. This was compared against methods previously used (relevant data was not available for all SNPs from the pre-determined samples), and results are detailed in Table 2. There was 100 % concordance between the array results and pre-determined genotypes for fourteen of the nineteen SNPs. For four of the remaining five SNPs (located in *APOE*, *APOB*, *CETP* and *LPL*) there was only one discordant result and for the fifth (located close to *MIA3*) there were two discordant results. Overall for the 19 genotypes the concordance rate was 3152/3158 = 99.8 %.

**Table 2:** Performance of the randox cardiac risk prediction array

SNP	Gene/Locus	Chromo-some	RAF in NPHSII	Genotype distribution			% Concordance (n)
				Common homozygotes (n)	Heterozygotes (n)	Rare homozygotes (n)	
rs11591147	*PCSK9*	1	0.99	139	5	0	100 (144)
rs17465637	*MIA3*	1	0.71	84	68	6	98.7 (158)
rs646776	*SORT1*	1	0.78	95	69	8	100 (172)
rs1042031	*APOB*	2	0.18	105	62	6	99.4 (173)
rs9818870	*MRAS*	3	0.16	116	44	5	100 (165)
rs3798220	*LPA*	6	0.02	166	7	0	100 (173)
rs10455872	*LPA*	6	0.07	135	20	1	100 (156)
rs1799983	*NOS3*	7	0.33	68	79	24	100 (171)
rs328	*LPL*	8	0.90	131	34	2	99.4 (167)
rs1801177	*LPL*	8	0.01	162	5	0	100 (167)
rs7025486	*DAB2IP*	9	0.26	94	69	10	100 (173)
rs10757274	9p21	9	0.48	57	84	33	100 (174)
rs1746048	*CXCL12*	10	0.86	129	41	4	100 (174)
rs662799	*APOA5*	11	0.06	133	13	2	100 (148)
rs17228212	*SMAD3*	15	0.31	83	72	18	100 (173)
rs708272	*CETP*	16	0.56	50	71	26	99.4 (158)
rs4341	*ACE*	17	0.52	47	81	40	100 (168)
rs7412	*APOE*	19	0.91	131	36	5	99.4 (172)
rs429358	*APOE*	19	0.17	139	32	1	100 (172)

*RAF* risk allele frequency from the sample of health UK men from Beaney et al. [9]. *SNP* single nucleotide polymorphism

**Table 3:** Association of gene score with T2D-CHD risk factors in the CoRDia SMI + RR study participants

Trait	Tertile 1 (n = 24)	Tertile 2 (n = 23)	Tertile 3 (n = 23)	p value
Age (years)	62.88 (6.96)	61.61 (8.15)	62.57 (7.38)	0.88
Sex (% female)	38 % (n = 9)	39 % n = 9	26 % n = 6	0.42
Ethnicity (% non Afro-Caribbean)	100 %	91 % (n = 21)	100 %	0.98
Total cholesterol (mmol/l)	4.26 (1.27)	4.58 (1.21)	4.42 (0.77)	0.63
HDL-cholesterol (mmol/l)^a^	1.21 (0.37)	1.12 (0.27)	1.17 (0.42)	0.50
Systolic blood pressure (mmHg)	129.38 (12.08)	137.91 (12.96)	134.43 (13.41)	0.18
Duration of T2D (years)^b^	5.28 (3.75–9.00)	5.79 (2.50–11.00)	8.12 (5.00–12.00)	0.08
Age of T2D Onset (years)	56.79 (7.80)	54.09 (9.63)	53.74 (7.58)	0.21
Smoking % (n)	4 % (n = 1)	22 % (n = 5)	22 % (n = 5)	0.11
Glycated haemoglobin (%)^a^	7.29 (4.92)	8.01 (8.70)	7.46 (9.98)	0.52

Gene score was divided into tertiles. Linear regression was performed for the numeric variables and logistic regression was performed for the categorical variables. Mean value and standard deviation for each tertile is shown except for log transformed variables were the geometric mean and approximate standard deviation are shown and square root transformed variables where means were transformed back and the range is shown

*HDL* high density lipoprotein, *T2D* type 2 diabetes, *CHD* coronary heart disease

^a^Variable was log transformed

^b^Variable was square root transformed

**Fig. 2:**
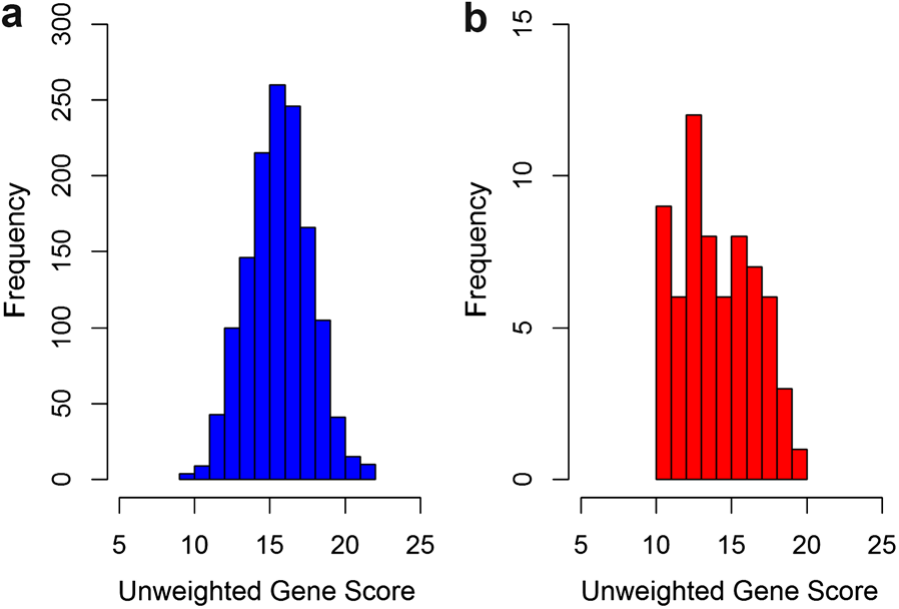
Distribution of risk alleles in **a** UK healthy men (n = 1360) and **b** CoRDia participants of Caucasian ethnicity in the SMI plus risk results arm (n = 66)*

**Fig. 3:**
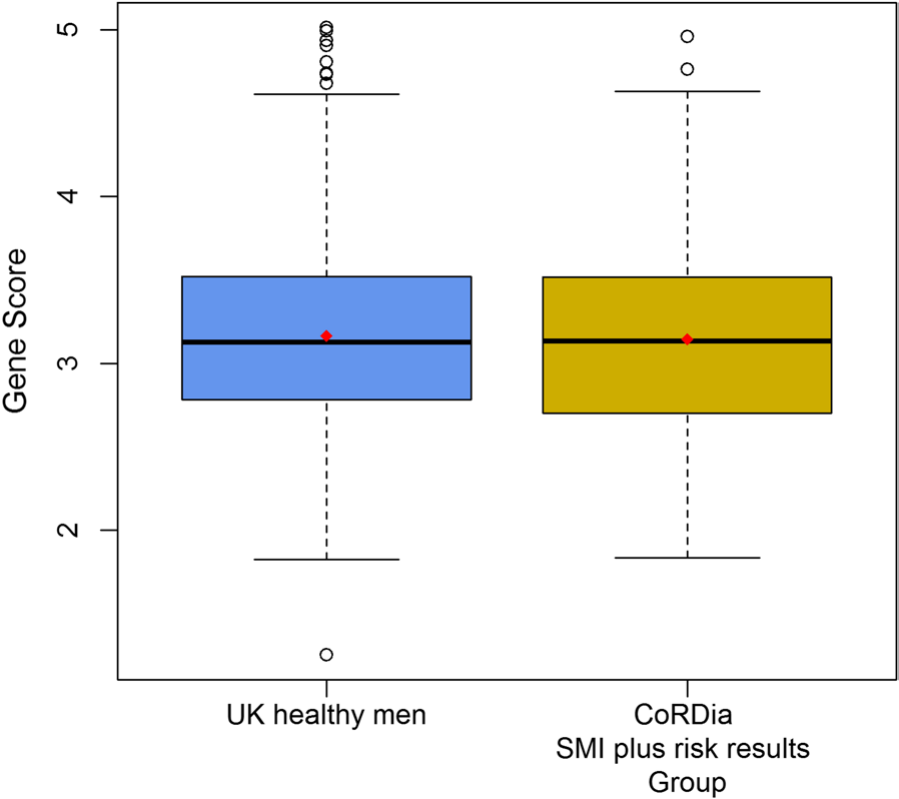
*Boxplot* of gene score in UK healthy men and the CoRDia SMI plus risk results arm. The mean weighted gene score for each group is marked in *red* (*horizontal line* represents the median) and these were compared using a *t* test (p = 0.80). Non-Caucasian participants were removed from the CoRDia SMI plus risk results profile group. The number of participants in each was as follows, NPHSII n = 1360 and CoRDia plus risk results n = 66

The genetic risk profile of the CoRDia recruits was compared to that of a cohort of healthy UK men, NPHSII. As the subjects in this group were all of Caucasian ethnicity, the analysis was performed after removal of the non-Caucasian participants from the CoRDia SMI + RR group. The distribution of risk alleles in the UK healthy men (n = 1360) and the Caucasian participants in the CoRDia SMI + RR group (n = 66 as there were four non-Caucasian participants in this group) is shown in Fig. 2. The range of risk alleles carried in the healthy men was 9-22 with a median number of 16. The range of risk alleles carried in the CoRDia SMI + RR group was 10–20, with a median number of 14. There was no significant difference in distribution of risk alleles between the two groups (as determined by Chi squared test, p = 0.53). When the gene score was calculated, weighting the carriage of the SNP for its reported CHD risk effect, there was no significant difference in mean gene score between the Caucasian participants of the CoRDia SMI + RR group and the group of healthy UK men (3.14 vs 3.17 p = 0.80, Fig. 3). The gene score was then combined with the UKPDS risk to give an overall ten-year risk of CHD. This ranged from 3–66 % (Fig. 1b).

To study the relationship between classical CHD risk factors and the gene score we divided the gene score into tertiles and performed regression analysis with the NPHSII data. The gene score was associated only with low density lipoprotein (LDL) cholesterol, with those in the top tertile of the score having 0.14 mmol/l higher levels than those in the bottom tertile (p = 0.05) and with family history of early CHD, with prevalence in the top tertile being 7 % higher than the bottom tertile (p = 0.03) (Table 4). For comparison, a similar analysis of the relationship between the gene score and T2D-CHD CRFs included in the UKPDS score is presented in Table 3 in the CoRDia SMI + RR group. In this small sample the gene score was not significantly associated with any of the variables assessed.

**Table 4:** Association of gene score with CHD risk factors in NPSHII

Trait	Tertile 1 (n = 454)	Tertile 2 (n = 455)	Tertile 3 (n = 451)	p value
Age (years)	56.45 (3.43)	56.17 (3.57)	56.51 (3.57)	0.86
Total cholesterol (mmol/l)	5.81 (0.98)	5.73 (1.02)	5.82 (0.98)	0.17
LDL-cholesterol (mmol/l)	3.10 (1.03)	3.18 (1.00)	3.24 (0.94)	0.05
HDL-cholesterol (mmol/l)	1.69 (0.58)	1.65 (0.58)	1.64 (0.59)	0.23
Triglyceride (mmol/l)^a^	1.77 (0.92)	1.82 (0.98)	1.86 (0.99)	0.19
Systolic blood pressure (mmHg)	139.88 (19.68)	138.61 (18.98)	139.66 (20.03)	0.86
Smoking % (n)	28 % (n = 128)	26 % (n = 119)	31 % (n = 140)	0.34
Family history of CHD % (n)	33 % (n = 139)	34 % (n = 145)	40 % (n = 169)	0.03
T2D onset % (n)	2 % (n = 8)	4 % (n = 17)	3 % (n = 12)	0.41
BMI (kg/m^2^)^a^	26.28 (3.20)	26.23 (3.37)	26.37 (3.48)	0.68

Gene score was divided into tertiles. Linear regression was performed for the numeric variables and logistic regression was performed for the categorical variables. Mean value and standard deviation for each tertile is shown except for log transformed variables were the geometric mean and approximate standard deviation are shown

*HDL* high density lipoprotein, *T2D* type 2 diabetes, *CHD* coronary heart disease, *BMI* body mass index

^a^Variable was log transformed

## Discussion

We have presented the baseline characteristics and genetic profile of the CoRDia study recruits, all of whom have T2D. The use of the T2D specific CHD risk calculator UKPDS, aims to take of account of the impact of diabetes management as well as its duration on risk of CHD, information which is not captured using risk models from the general population. The range UKPDS ten-year CHD-risk score observed in the CoRDia recruits of 2–72 % is similar to that observed in the general population with T2D [15] and to the Caucasian participants of the University College London Diabetes and Cardiovascular Disease Study (UDACS) [16] where the range of UKPDS 10-year CHD-risk scores in the Caucasian participants without CHD was 1–87 %. Furthermore, the UKPDS risk scores did not differ significantly between the three study groups demonstrating successful randomisation of the participants.

The use of saliva as a sample matrix proved effective, providing a high yield of good quality DNA, demonstrated by its use with both the Cardiac Risk Prediction Array and as template DNA for Sanger sequencing. A further benefit was that samples, once collected, are stable, easily stored and could be posted to the genetics lab. Furthermore, the Cardiac Risk Prediction Array showed very high concordance with the other genotyping methods used (99.8 %). A number of the SNPs included in the gene score have low minor allele frequencies (MAF), consequently few samples with these alleles were available for testing and thus confidence intervals for the concordance values given are large. Three of the 19 SNPs, rs3798820 in *LPA*, rs11591147 in *PCSK9* and rs1801177 in *LPL* have MAFs below 0.05 in NPHSII. As such, we were only able to test a few heterozygous samples and no rare homozygous samples for these SNPs. However, it must be noted that the Cardiac Risk Prediction array results for the SNPs *LPA* rs10455872 and *APOA5* rs662799, which have similarly low MAFs, were in complete concordance with the other genotyping methods used. The SNP rs17456537, close to the gene *MIA3* on chromosome 1, was the only SNP to have more than one discordant result between the Cardiac Risk Prediction array and the pre-determined genotype for the blood-extracted DNA. While the molecular principles underlying the Cardiac Risk Prediction array have been used in other products, this is the first to use for genotyping. Given the high concordance between the array and other “gold-standard” methods such as TaqMan, we can be confident that the genotyping was accurate and thus the gene score calculated on the basis of this was a true reflection of the participant’s genetic CHD risk.

The distribution of risk alleles in the sample healthy UK men can be seen to be normally distributed. While in the CoRDia SMI + RR group the distribution does not show the same classic “bell-curve” and normally distributed characteristics, this can be attributed to the relatively low number of participants. As there was no significant difference in the mean gene score of the genotyped CoRDia recruits and the large cohort of healthy UK men, we can conclude that the genetic CHD risk observed in our cohort is typical of the UK population, and therefore, we believe that the results of the study can be extrapolated to the general population, for example in the reaction of the participants to their genetic risk. While the CorDia subjects would be expected to have inherited a greater than average number of T2D risk alleles, the similar mean score for CHD risk alleles between the diabetic group and the UK healthy subjects is in line with GWAS studies that show that the genetic architecture of CHD and T2D are different, demonstrating by the fact that few of the CHD risk and T2D risk loci overlap [17, 18].

Both the SNPs included in the gene score and the weights of CHD risk associated with carriage of each risk allele [9] were obtained from studies with Caucasian participants. While the results of such studies will not reflect the underlying genetic architecture of CHD across all ethnicities, this is by far the largest source of data. Therefore and especially given that over 90 % of participants of the CoRDia study are Caucasian, the use of the 19 SNP gene score is appropriate for this cohort. Furthermore, while we accept that relatively small sample size is a limitation of the study, the use of the combined weighted gene score as the single major genetic variable examined, overcomes the potential problem of low power resulting from the inclusion of rare variants within the 19 SNPs. While many more SNPs have now been identified to be associated with risk of CHD by GWAS and the meta-analysis of these data [17], the 19 SNP CHD gene score used here has been previously found to be associated with CHD in subjects of both Caucasian [9] and Afro-Caribbean ethnicity [10], although the relationship with CHD in those of South Asian ethnicity requires further clarification [9]. Addition of further SNPs may improve the utility of such SNP scores in the future [19, 20].

In large part, the gene score captures information not included as part of the assessment of CHD risk by the UKPDS score. In the healthy UK subjects the score was significantly associated with higher LDL-cholesterol, which reflects the inclusion of SNPs in several genes involved in LDL-cholesterol metabolism (*APOB, PCSK9, APOE, CETP*) and, not surprisingly for a genetic instrument, with higher family history of CHD, which has previously been shown to be an independent risk factor for CHD in this cohort [12] and many others [21, 22]. However, the score was not significantly associated with other CHD or T2D-specific risk factors (total cholesterol, HDL-cholesterol, age, smoking, systolic blood pressures, BMI and risk of diabetes). In the smaller CoRDia SMI + RR cohort, the gene score was not significantly associated with any of the CHD-risk variables included in the UKPDS score, indicating that the gene score is capturing information concerning CHD risk over and above that captured by the UKPDS score. Therefore, inclusion of the gene score in risk prediction may improve the performance of the UKPDS risk tool alone. Since the composition of the gene score was finalised dozens of robustly association CHD risk loci have been identified primarily by the CARDIoGRAM consortium [23]. Inclusion of these variants in the gene score may improve its performance and provide a more accurate measure of an individual’s genetic risk of CHD, although the use of such gene score has given mixed results [19, 20].

In summary, baseline 10-year CHD risk did not differ significantly between the randomisation groups. We found the Randox Cardiac Risk Prediction Array results to be over 99.8 % concordant with other genotyping methods and used it to successfully genotype the SMI + RR arm of the CoRDia study. The gene score was modestly associated with effects on two well-known CHD risk factors, namely LDL-cholesterol and family history of CHD, but as was expected, there was no evidence of a higher gene score in the diabetic subjects compared to healthy UK subjects, confirming the lack of bias in recruitment.

## Abbreviations

CHD: coronary heart disease
CRF: conventional risk factor
CoRDia: coronary heart disease risk in type 2 diabetes
GWAS: genome-wide association study
hba1c: glycated haemoglobin
HDL: high density lipoprotein
LDL: low density lipoprotein
MAF: minor allele frequency
NPHSII: Northwick park heart study II
SMI: self-management intervention
SMI + RR: self-management intervention plus risk results
SNP: single nucleotide polymorphism
T2D: type 2 diabetes
